# Gene Ranking of RNA-Seq Data via Discriminant Non-Negative Matrix Factorization

**DOI:** 10.1371/journal.pone.0137782

**Published:** 2015-09-08

**Authors:** Zhilong Jia, Xiang Zhang, Naiyang Guan, Xiaochen Bo, Michael R. Barnes, Zhigang Luo

**Affiliations:** 1 Department of Chemistry and Biology, College of Science, National University of Defense Technology, Changsha, Hunan, P.R. China; 2 William Harvey Research Institute, Barts and The London School of Medicine and Dentistry, Queen Mary University of London, London, United Kingdom; 3 Science and Technology on Parallel and Distributed Processing Laboratory, College of Computer, National University of Defense Technology, Changsha, Hunan, P.R. China; 4 Beijing Institute of Radiation Medicine, Beijing, P.R. China; Georgia Institute of Technology, UNITED STATES

## Abstract

RNA-sequencing is rapidly becoming the method of choice for studying the full complexity of transcriptomes, however with increasing dimensionality, accurate gene ranking is becoming increasingly challenging. This paper proposes an accurate and sensitive gene ranking method that implements discriminant non-negative matrix factorization (DNMF) for RNA-seq data. To the best of our knowledge, this is the first work to explore the utility of DNMF for gene ranking. When incorporating Fisher’s discriminant criteria and setting the reduced dimension as two, DNMF learns two factors to approximate the original gene expression data, abstracting the up-regulated or down-regulated metagene by using the sample label information. The first factor denotes all the genes’ weights of two metagenes as the additive combination of all genes, while the second learned factor represents the expression values of two metagenes. In the gene ranking stage, all the genes are ranked as a descending sequence according to the differential values of the metagene weights. Leveraging the nature of NMF and Fisher’s criterion, DNMF can robustly boost the gene ranking performance. The Area Under the Curve analysis of differential expression analysis on two benchmarking tests of four RNA-seq data sets with similar phenotypes showed that our proposed DNMF-based gene ranking method outperforms other widely used methods. Moreover, the Gene Set Enrichment Analysis also showed DNMF outweighs others. DNMF is also computationally efficient, substantially outperforming all other benchmarked methods. Consequently, we suggest DNMF is an effective method for the analysis of differential gene expression and gene ranking for RNA-seq data.

## Introduction

RNA-sequencing (RNA-seq) is a powerful transcriptome analysis method to identify differential transcript expression between different biological states. It is commonly considered superior to fixed transcriptome analysis platforms such as microarrays, however the complexity of the data generated by the technique presents some inherent challenges to accurate interpretation. Generally, a list of differentially expressed genes will be obtained using an arbitrary threshold (such as a P-value < 0.05 after correction for multiple testing). A statistical false discovery threshold can be used to identify those genes most likely related to a given phenotype [1]. But this strategy inevitably leads to a loss of information, which could otherwise inform on the full complexity of a biological mechanism or process. An alternative approach to tackle this problem, is to rank genes in a logical order of differential expression, rather than on the basis of a somewhat arbitrary statistical threshold [2].

Implementation of an accurate gene ranking is an important prior step to downstream analysis, such as Gene Set Enrichment Analysis (GSEA), which is more likely to result in biologically meaningful insights into the underlying biology of the condition or process being studied. GSEA provides some built-in metrics for ranking genes produced in microarray experiments, such as Signal2Noise, tTest, and fold change for categorical phenotypes (see GSEA user guide). For RNA-seq data, GSEA recommends to use other RNA-seq related differential expression analysis tools to rank genes. After obtaining the rank of genes, the user can use GSEAPreranked in GSEA to continue the analysis.

A number of tools have been developed to enable differential expression analysis of RNA-seq data, such as DESeq [3], edgeR [4], PoissonSeq [5] and gfold [6]. However, their analytical results usually differ according to the underlying differential expression algorithms applied [7]. To the best of our knowledge, gfold is the only tool which offers a specific capability to rank genes from RNA-seq data, while other differential expression analysis tools tend to focus on finding differentially expressed genes. The gfold is a generalized fold change based on the posterior distribution of log fold change for ranking differential genes from RNA-seq data. In general, biologists prefer to rank genes according to P-values produced by differential expression analysis tools, but P-values may not be the most effective gene ranking index. In general it is unclear which GSEA ranking method is most appropriate for RNA-seq data.

Nonnegative matrix factorization (NMF), proposed in 1999 by Lee and Seung, has been successfully applied in both face recognition and text mining [8]. NMF is an unsupervised, parts-based representation and dimension reduction paradigm, which decomposes a nonnegative matrix V into two lower-rank non-negative matrices, i.e., V~WH, via a multiplicative update rule (MUR) [8]. NMF has been in the ascendant since it was first described. It offers several innate advantages, including an intuitive interpretation of factorization and an implicit sparse representation of complexity that is well suited to the identification of prominent features [9]. In addition, with the incorporated non-negativity constraints over factor matrices, NMF is distinguished from principal component analysis (PCA) and singular value decomposition (SVD) [9]. There are plentiful applications of NMF and its extension to data rich fields, including neural computing, pattern recognition, signal processing, spectral data analysis, chemometrics, geophysics and more recently computational biology, such as molecular pattern discovery, class comparison and prediction, cross-platform and cross species analysis, functional characterization of genes and biomedical text mining [9–15]. Among the biological applications to date, NMF has been successfully used for differential expression analysis of microarray data [11]. However, the undirected nature of the method has one drawback, as NMF performs clustering without leveraging the sample label information. Although this approach may possess advantages in poorly defined phenotypes, prediction performance may be poorer than direct sample assignment where phenotypic characterisation is precise, which limits NMF as a general tool for differential expression analysis or gene ranking. The application of NMF to differential expression analysis of RNA-seq data has not been specifically explored, although NMF has been proposed as a method for normalizing RNA-seq data [16].

By integrating discriminant terms into NMF, discriminant NMF (DNMF) was developed by regarding Fisher’s discriminant criterion, i.e., the difference between the within-class scatter and the between-class scatter, as a penalty term of DNMF [17]. To increase the free degree of the model parameter, Zafeiriou et al proposed a more flexible DNMF, applied to frontal face verification [18]. Kotsia et al solved DNMF based on projected gradient method, which is more suitable for classification tasks [19]. Recently, Lee et al, developed a variant of DNMF, improving discriminant power by using the trace of between-class scatter and then applied to both emotion and speech recognition [20, 21]. Collectively, DNMF not only improves the separation between different classes by the discriminant Fisher’s criterion but also learns the sparse representation inherited from NMF.

Here we apply DNMF to gene ranking of RNA-seq data. DNMF is distinct from NMF, by assigning rather than predicting sample labels. Thus, up-regulated and down-regulated metagenes may be more predictive than those generated by NMF. When applied to gene ranking of RNA-seq data, DNMF provides a more efficient and flexible approach to differential expression analysis as well as gene ranking, and is computationally more efficient compared with other routinely used methods.

## Methods

### RNA-seq test datasets

We used four well-characterized reference RNA-seq datasets (AGR, BGI, NWU, PSU) with a set of synthetic RNAs from the External RNA Control Consortium (ERCC) at known concentrations derived from the Sequencing Quality Control (SEQC) project (GSE47792) [22]. Two sample groups, A and B, were selected. Sample group A is composed of total RNA from ten human cell lines, derived from Agilent’s Universal Human Reference RNA (UHRR), while sample group B is from Life Technologies’ Human Brain Reference RNA (HBRR) cell lines, pooled from multiple donors and several brain regions. Sample group A and B were mixed with Ambion ERCC RNA Spike-In Mix 1 and 2 accordingly. The two ERCC RNA mixtures in groups A and B contain various concentrations of four subgroups of the synthetic spike-ins with a predefined log fold change, resulting in 93 ERCC RNA as a benchmark for evaluating differential expression analysis.

The AGR dataset and BGI dataset were sequenced by the Illumina HiSeq 2000 platform, while the NWU dataset and PSU dataset were sequenced by the Life Technologies’ SOLiD 5500 platform. All the libraries were mapped to the GRCg37/hg19 human genome by using the Subread package [23]. Raw read counts of the four datasets were obtained from the Bioconductor package seqc [22]. Technical replicates sequenced across different lanes and flow cells were combined for each sample, as the impact of technical variation is likely to be minor compared to variation introduced by library preparation or biological variation. Transcripts are represented by raw read counts in all datasets (see Table 1). To evaluate evidence for differential expression, the TaqMan qPCR dataset, containing 1044 selected genes, was used to validate differential transcript expression. Considering the high quality nature of the SEQC dataset and the availability of qPCR validation data, we prioritised this above other, more heterogeneous publically available RNA-seq datasets without qPCR validation data.

**Table 1:** Description of four RNA-seq datasets.

dataset	Platform	#reads	#transcripts	#samples	#libraries
AGR	Illumina	949945481	24550	8	256
BGI	Illumina	657087509	24550	10	384
NWU	SOLiD	357571839	24550	10	288
PSU	SOLiD	284509053	24550	10	288

#reads: total number of sequence reads; #transcripts: total number of transcripts; #samples: total number of samples and #libraries: total number of libraries sequenced.

### Gene ranking

Five methods for gene ranking are compared with DNMF. As P-value and log fold change (LogFC) is often used to evaluate significant results from differential expression analysis and the up-regulated and down-regulated genes are usually at the top and bottom of the ranked gene list, respectively, we use the signed P-value to rank genes, where the sign is from LogFC. Genes with same P-value are ranked based on LogFC. We compare the ranking methods of the signed P-value from DESeq (DESeq, version 1.16.0), the signed P-value from edgeR (edgeR, version 3.6.8), the gfold value based method (gfold, version 1.13), the LogFC from gfold based method (gfoldFC), the tt score statistic from PoissonSeq (PoissonSeq, 1.1.2) and DNMF. For DESeq and edgeR, the built-in methods are used to normalize the data prior to DE analysis [24], and the size factor normalization method from DESeq is applied for DNMF, while gfold utilizes the read count and transcript length directly. Additionally, normalized counts are internally log2 transformed after addition of pseudo counts for the DNMF method.

### Discriminant Non-negative Matrix Factorization (DNMF)

Non-negative Matrix Factorization approximates the original data *V* by the product of both factors, i.e. *W* and *H*, Thus, NMF can be written as follows:

V≈WH. (1)

To measure the approximate error (or noise) of (1), we can resort to different distance definitions such as the Euclidian Distance and Kullback-Leiblur (KL) divergence. Different distance metrics correspond to different objective functions and reflect the statistical structure of the original data and the disclosed components. For instance, the Euclidian Distance can identify the Gaussian noise while the KL divergence can model the Poisson distribution of the data noise. Here, we employ the KL divergence [25] to evaluate the approximate error in DNMF [18].

When applied to gene ranking, the gene expression profile is represented by matrix *V* with gene in row and sample in column. The objective of DNMF is to minimize the KL divergence between *V* and the product of both *W* and *H* as follows:

$$D_{KL}\left(V∥WH\right)=\sum_{il}\;V_{il}log\frac{V_{il}}{{\left(WH\right)}_{il}}-V_{il}+{\left(WH\right)}_{il}$$ (2)

where *V*_*il*_ is the row (gene) *i* and column (sample) *l* in matrix *V*, *W* is a matrix with gene in row and metagene in column and *H* is a matrix with metagene in row and sample in column.

The vector *h*_*j*_ (the *j*-th column (sample) of the matrix *H*), is the coefficient vector for the *ρ*-th sample of the *r*-th class (e.g. the control group and treatment group) and denoted as

$$\eta_{\rho }^{\left(\text{r}\right)}={\left[\eta_{\rho ,1}^{\left(\text{r}\right)}\cdots \eta_{\rho ,k}^{\left(\text{r}\right)}\cdots \eta_{\rho ,K}^{\left(\text{r}\right)}\right]}^{T},$$ (3)

where *k* is the *k*-th row (metagene) in H.

The mean of the vectors $\eta_{\rho }^{\left(\text{r}\right)}$ for the class r is denoted as

$$\mu^{\left(\text{r}\right)}={\left[\mu_{1}^{\left(\text{r}\right)}\cdots \mu_{k}^{\left(\text{r}\right)}\cdots \mu_{K}^{\left(\text{r}\right)}\right]}^{T},$$ (4)

while the mean vector of all the classes of samples as

$$\mu ={\left[\mu_{1}\cdots \mu_{k}\cdots \mu_{K}\right]}^{T}.$$ (5)

DNMF incorporates Fisher’s criterion into NMF by maximizing the distance among any samples from different classes meanwhile minimizing the dispersion between any pairs of samples in the same class. Thus, we define the within-class scatter matrix and between-class scatter matrix. According to (3), (4) and (5), the within-class scatter for the coefficient vectors *h*_*j*_ can be defined as

$$S_{w}=\sum_{r=1}^{R}\sum_{\rho =1}^{Nr}(\eta_{\rho }^{\left(\text{r}\right)}-\mu^{\left(\text{r}\right)}){\left(\eta_{\rho }^{\left(\text{r}\right)}-\mu^{\left(\text{r}\right)}\right)}^{T},$$ (6)

where *R* is the number of sample classes and *Nr* is the number of samples in the *r*-th class. and the between-class scatter matrix as

$$S_{b}={\sum_{r=1}^{R}Nr(\mu^{\left(\text{r}\right)}-\mu )(\mu^{\left(\text{r}\right)}-\mu )}^{T}.$$ (7)

The matrix *S*_*w*_ represents the scatter of samples within the same classes and its trace should be as small as possible; Meanwhile, the matrix *S*_*b*_ denotes the scatter of samples between the classes and its trace should be as large as possible. Thus, we can obtain the objective of DNMF as follows:

$$\sum_{il}V_{il}\;\text{log}\frac{V_{il}}{{\left(WH\right)}_{il}}-V_{il}+{\left(WH\right)}_{il}+\gamma \text{tr}[S_{w}]-\delta \text{tr}[S_{b}],$$ (8)

Where *γ* and *δ* are relative weighting factors and *tr* means trace.

To solve DNMF, Zafeiriou *et al*. proposed a multiplicative update rule (MUR) for both *W* and *H* [18]. The update rule for *H* is

$$h_{k,l}^{t}\leftarrow -\frac{b}{2a}+\frac{\sqrt{b^{2}-4ac}}{2a},$$ (9)

where $a=2\gamma +\frac{2\sigma }{L}-\frac{2\gamma +2\sigma }{N_{r}}$, $b=1+\frac{2\sigma }{L}\sum_{\begin{array}{l} j=1\\ j\neq l \end{array}}^{L}h_{kj}-\frac{2\gamma +2\sigma }{N_{r}}\sum_{\begin{array}{l} \lambda =1\\ \lambda \neq l \end{array}}^{Nr}h_{k,\lambda }^{t-1}$, $c=-\sum_{i}v_{i,l}\frac{w_{i,k}h_{{}_{k,\lambda }}^{t-1}}{\sum_{n}w_{i,n}h_{n,l}^{t-1}}$, wherein $h_{k,l}^{t}$ is the *k*-th metagene and *l*-th sample element in *t*-th iteration and *L* is the column number of *H* (the number of samples).

The MUR for *W* is the same as that of NMF, i.e.

$$w_{i,k}\leftarrow w_{i,k}\sum_{l}\frac{v_{i,l}}{{\left(WH\right)}_{i,l}}h_{k,l},$$ (10)

$$w_{i,k}\leftarrow \frac{w_{i,k}}{\sum_{l}w_{l,k}},$$ (11)

where (11) implements a normalization step and *w*_*i*,k_ means the *i*-th gene and *k* metagene element of *w*.

The detailed derivation is given in [18]. In the formulation (38) of [18], the variable *μ*_*k*_ should be substituted by

$$\mu_{k}=\frac{1}{L}\sum_{j=1}^{L}h_{kj}$$ (12)

since it actually includes this entry, i.e., *h*_*kj*_.

According to (9), (10) and (11), we implement DNMF using R, which is available at CRAN (http://cran.r-project.org/web/packages/DNMF). In this implementation, we initiate W and H randomly and then add the total sum of each element in *H* to the *k*-th metagene within the *r*-th (*r = k*) class. This step makes DNMF more stable and results in clearly defined up-regulated and down-regulated metagenes in *H*. In the case of *k = 2* and the control is the first sample group, the first row of *H* is the down-regulated metagene, while the second the up-regulated metagene. Formally, the down-regulated metagene will be the *k*-th row of *H* if $\mu_{k}^{\left(1\right)}>\mu_{k}^{\left(2\right)}$, while the up-regulated metagene will be the *k*-th row of *H* if $\mu_{k}^{\left(1\right)}<\mu_{k}^{\left(2\right)}$.

Because of the correspondence between the columns of *W* and the rows of *H*, the first column of *W* is the linear combination of down-regulated genes, while the second column of *W* is that of up-regulated genes. The *W* represents the weight of each genes in the two regulated metagenes. Consequently, the rank of genes can be defined as

$$d=W_{2}-W_{1},$$ (13)

Where *W*_*1*_ and *W*_*2*_ are the first and second column in *W*, and the sign of *d* indicates the regulation direction.

**Fig 1:**
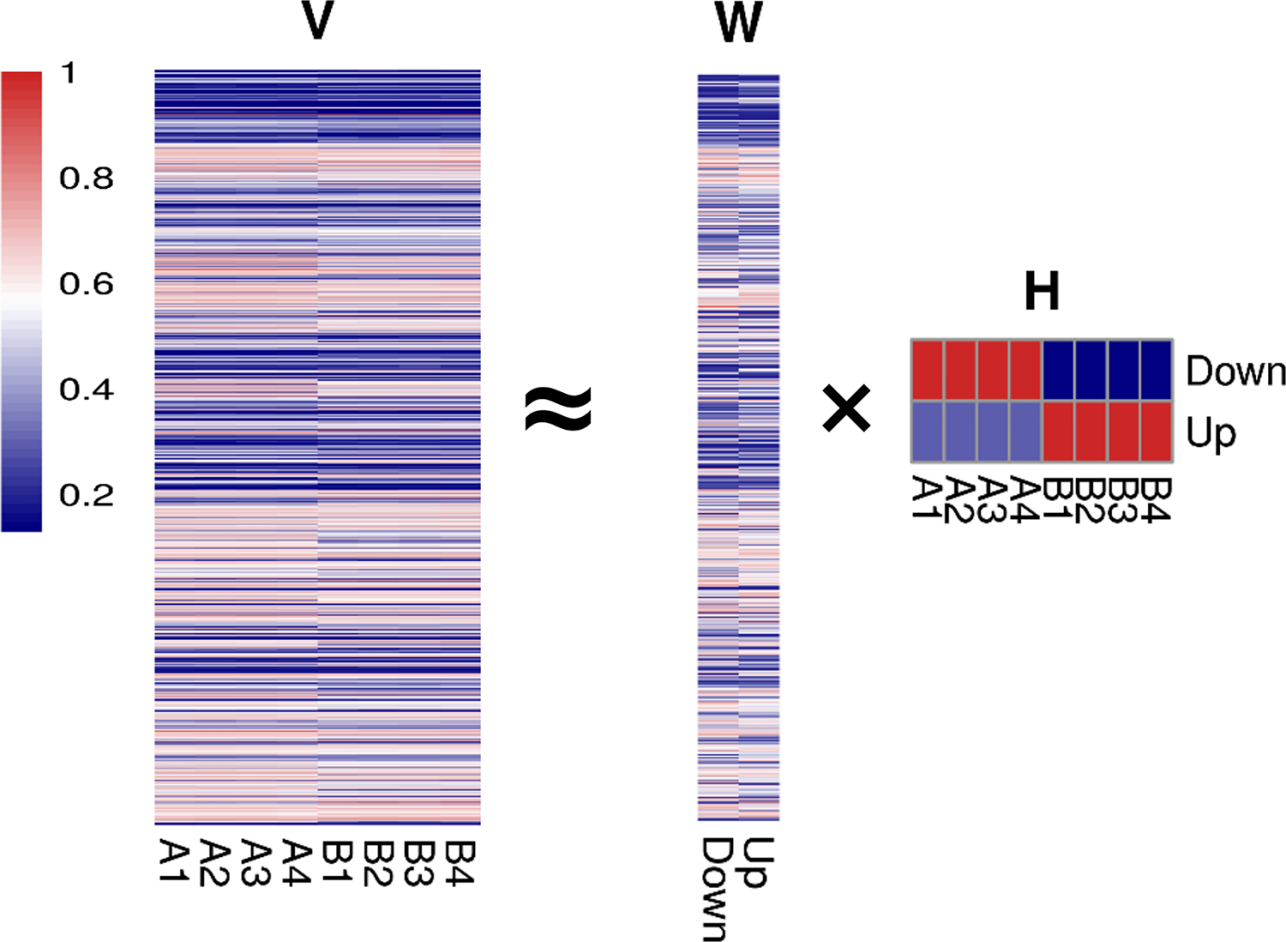
Decomposition of RNA-seq data by DNMF. There are two types of samples, A and B, in the matrix *V*, each with four replicates. Via DNMF, two matrices, *W* and *H*, are produced. The matrix *H* possesses the same column number as matrix *V*, while the matrix *W* possesses the same row number as matrix *V*. To explain it biologically, the row of *H* could be viewed as the metagenes (the up-regulated or down-regulated gene sets), while the column of *W* represents the weight of each gene in the metagenes. The two metagenes in matrix *H*, represent the up-regulated genes and down-regulated genes respectively. Due to the correspondence between columns of *W* and rows of *H*, it is possible to identify which metagenes are combined with the up-regulated or down-regulated gene sets in matrix *W*. Consequently, it is easy to obtain the rank of genes by subtraction between the weights of the two metagenes in matrix *W*. The scaled AGR dataset is used in this figure.

When applied to gene expression data, DNMF is easily interpretable. Given gene expression data for *n* genes in *m* samples, we represent the data as a *n×m*-dimensional matrix *V*, wherein each entry represents the gene expression of each sample. The decomposed matrix *W* of size *n×k* represents the gene weight in metagenes, while *k×m*-dimensional *H* denotes the metagene expression in sample and the metagene is defined as a nonnegative linear combination of the genes [10]. With an appropriate cost function and Fisher’s criterion, this type of decomposition can substantially increase the signal to noise ratio of a dataset. Considering the gene ranking, setting the *k* to two will extract the most canonical two metagenes with the Fisher’s criterion. DNMF tries to make the two rows of *H* largely different to capture two metagenes representing the up regulated and down regulated genes, as showed in Fig 1. We can determine which metagene represents the up-regulated metagene or the down-regulated metagene in the two rows of matrix *H* based on the values. Due to the correspondence between the columns of *W* and the rows of *H*, we can determine which metagene is the up-regulated or down-regulated metagene in the two column of matrix *W*. The up-regulated (or down-regulated) metagene is the linear combination of all the genes with the value representing the weight of as up-regulated genes (or down-regulated). In other words, the metagene can be viewed as a combination of highly weighted differentially expressed genes and low or zero weighted non- differentially expressed genes. Consequently, it is easy to obtain the rank of up-regulated or down-regulated genes based on the values (weights) in matrix *W*. Finally, we can get the rank of genes by the subtraction between the weights of two metagenes in matrix *W*. We set two parameters, i.e., gamma and delta in formula (9), to 0.1 and 0.0001, respectively.

A permutation test is used to estimate the significance of differentially expressed genes, based on a test implemented by Wang et al [26]. The null hypothesis is that matrix *W* is non-discriminative of the sample classification. The permutated *d*_*s*_ can be obtained by shuffling the elements of *W B* = 1000 times randomly and then subtracting between two columns of *W*. accordingly, the P-value for an observed d will be

$$p=\frac{1}{nB}\sum_{b=1}^{B}\sum_{i=1}^{n}I(|d|<|d_{i}^{b}|)\;,$$ (14)

Where *I*(⋅) is an indicator function, being 1 if true and 0 otherwise, *n* is the number of genes, and $d_{i}^{b}$ is the permuted d for gene *i* in the *b*-th permutation.

### Gene set enrichment analysis (GSEA)

Gene set enrichment analysis is a ranking based gene set enrichment method [2]. Since these ranking results are not from a built-in GSEA function, we use the GSEAPreranked tool of GSEA (version 2.1.0) directly. The C2 Canonical pathways (C2CP), C2 KEGG (C2KEGG), and C5 GO biological process (C5) gene datasets from the Molecular Signatures Database [2] are used as the test gene datasets. There are 1320 gene sets in the C2CP gene dataset, 186 gene sets in the C2KEGG gene dataset and 825 gene sets in the C5 gene dataset. The classic scoring scheme, which penalizes sets for lack of coherence and is the only available scoring scheme for a pre-ranked gene list, the gene set permutation type and other default parameters were used.

### Evaluation Criteria

Three aspects are compared to evaluate the performance of different methods. Firstly, The Area Under the Curve (AUC) benchmarked by 93 pre-defined ERCC spike-ins is compared. Three thresholds (0.58, 1 and 2) for absolute logFC are set up. Secondly, with increasing absolute logFC from 0.5 to 2 (incremented by 0.1), the AUC benchmarked by 1044 qPCR validated genes, is compared too. The pROC package [27] is used for calculating the AUC. Finally, because sample B in the SEQC project is brain-related among all four datasets, the agreement of enriched gene sets among all four datasets based on the top 10 enriched gene sets of sample B for each ranking methods are evaluated and the overlapping enriched gene sets are compared. The code of the full analytic process is available at https://github.com/zhilongjia/geneRanking.

## Results

We compared the performance of a range of different ranking methods. Specifically, we studied six RNA-seq expression ranking methods, DESeq, DNMF, edgeR, PoissionSeq, gfold and gfoldFC. Results were compared using canonical pathway (C2CP) gene datasets, KEGG (C2KEGG) and GO process (C5) gene datasets, respectively. All the detailed results are also supplemented with the analytic code.

### Comparison of MA plots

**Fig 2:**
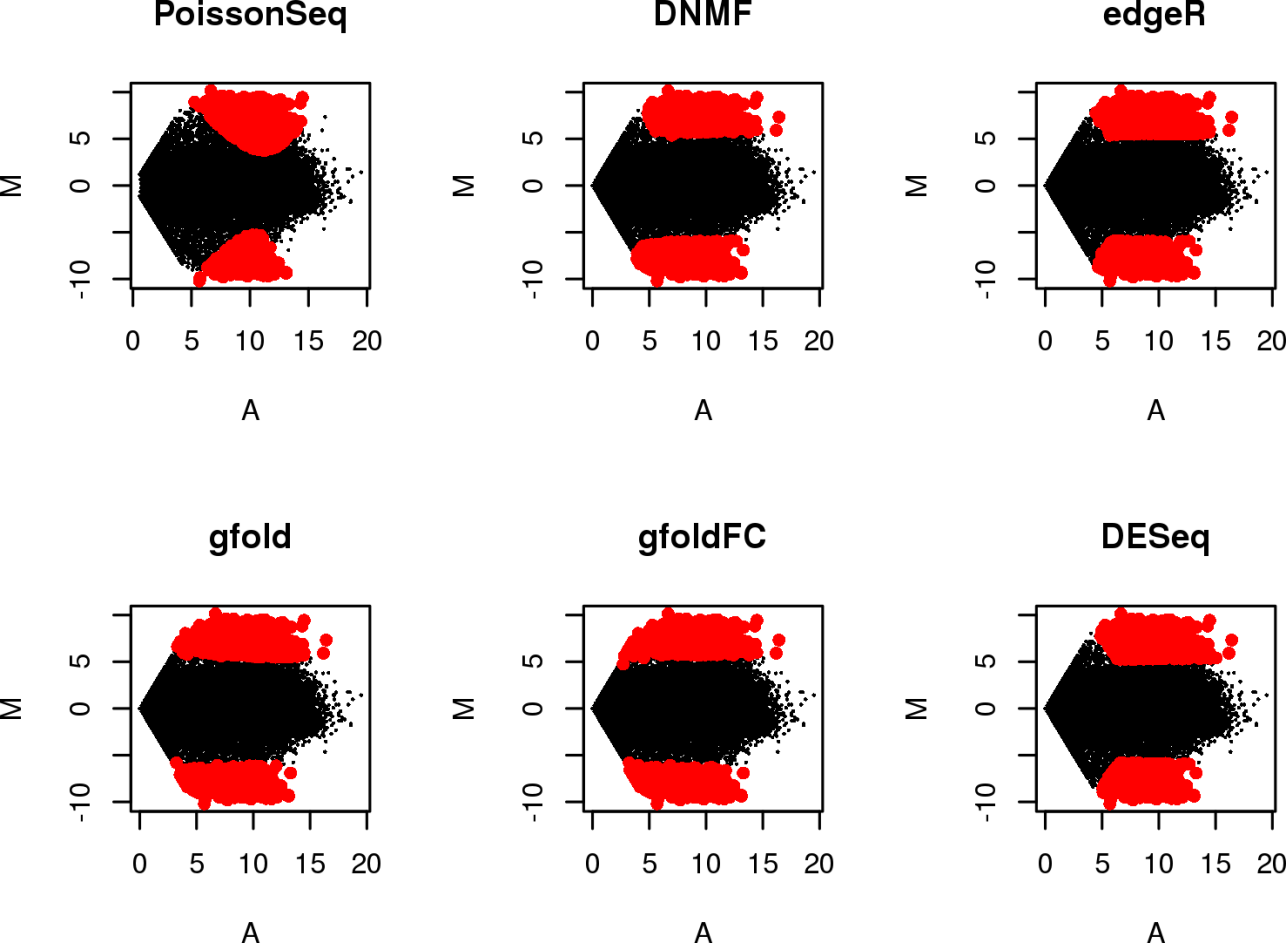
MA plot of top 1000 differentially expressed genes in the AGR dataset identified by 6 methods. The means of two different kinds of samples in the data are used. Red dots indicate differentially expressed genes. Black dots indicate non-differentially expressed genes.

MA plots are a direct and convenient way to show the distribution of differentially expressed genes among all genes and the inherent bias of gene ranking methods on the basis of expression level. The distribution of variance and signal strength of the top 1000 differentially expressed genes identified by all six methods on the AGR dataset are shown in Fig 2. The results on the remaining three datasets are shown in S1–S3 Figs. As shown in Fig 2, DNMF had less preference for genes with larger expression when compared to edgeR and DESeq, though gfold and gfoldFC performed best among them. However, compared to gfold and gfoldFC, DNMF preferred with medium expressed genes than very low expressed genes. We believe this bias is desirable because the logFC of genes with very low expression is more prone to error and can often lead to confounding outliers in gene expression analysis. The agreement of enriched gene sets illustrates that this kind of bias is desirable in the analysis of differential gene expression. In addition, in a similar manner to DESeq and edgeR, DNMF chose up-regulated and down-regulated genes more equally than gfold, gfoldFC and PoissonSeq (S4 Fig). The top 10 genes on the other three datasets showed similar behaviour (data not shown).

### Comparison of differential expression analysis based on ERCC data and qPCR validated data

**Fig 3:**
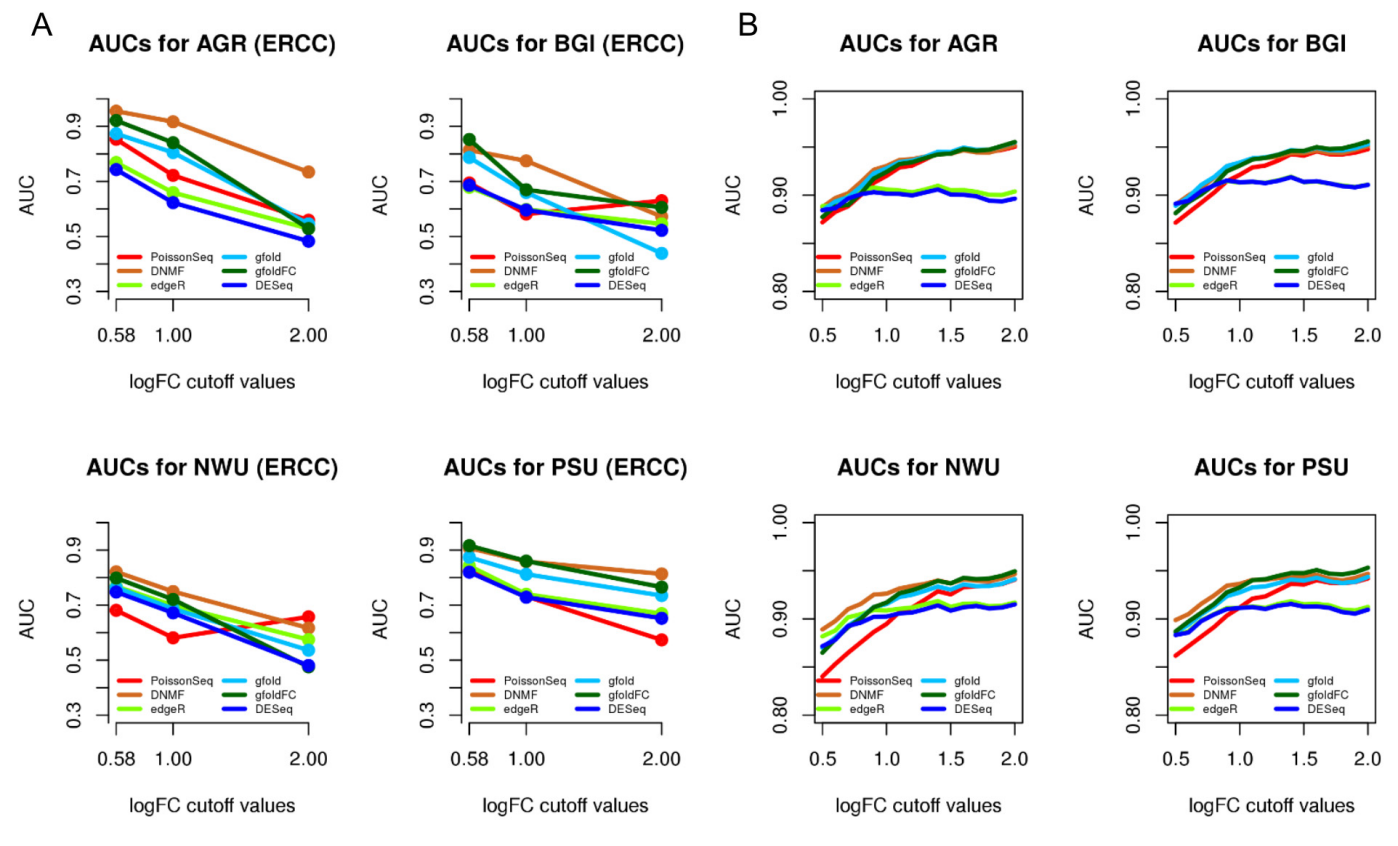
AUC curve of differential expression analysis using ERCC and qPCR validated data. (A) The cutoffs of absolute log fold change (LogFC) (0.58, 1 and 2) are used. DNMF works best in most cases. (B) At increasing LogFC, the performance of DNMF and gfold shows a slight advantage over other methods, while at lower logFC thresholds, DNMF generally outperformed other methods. AUC, area under the curve.

We evaluated the ability of the six methods to detect differentially expressed genes based on ERCC data, representing genes with a range of LogFCs. Accordingly, it is possible to test how well all the tested methods correctly detect the extent of differential expression. ERCC with absolute LogFC more than 0.58, 1 and 2 are considered as differentially expressed, while the rest are non-differentially expressed. We performed an AUC analysis to compare the performance of all six methods in identifying differentially expressed ERCC (Fig 3A). Overall, DNMF outperformed the other methods in most situations, though gfoldFC worked comparably in some situations. Collectively, DNMF is the optimum method for differential expression analysis of RNA-seq data in this comparison.

Due to the limited number of ERCC spike-ins available, we further tested the performance of the six methods based on a more comprehensive benchmarked dataset with qPCR validation, spanning a wider range of LogFC and sampling a wider transcript population [28]. We measured AUC at increasing cutoffs of log2 qPCR expression changes, defining differentially expressed genes as genes above the absolute LogFC. The results are shown in Fig 3B. Generally, all methods performed comparably on all the four datasets with a slight advantage of gfoldFC and DNMF. However, DNMF outperformed other methods at lower logFC thresholds.

### Comparison of enriched gene set agreement among all datasets

It is one of the fundamental goals of GSEA to obtain a consistent signal from independent datasets concerning the same study objective or phenotype. Accurate identification of a true signal, overcoming the confounding influence of biological and experimental noise is the acid test of gene set enrichment analysis. Because sample B is derived from multiple brain regions, we performed GSEA based on three gene datasets (C2CP, C2KEGG and C5) on all four expression datasets and then investigated the agreement of enriched gene sets related to sample B between all four expression datasets using the top 10 enriched gene sets (more details shown in Table 2). From Table 2, the largest number of overlapping C2CP gene sets are 9/10, obtained by DNMF and gfoldFC methods. The edgeR and DNMF methods showed 9/10 gene overlap among all four datasets on the C2KEGG gene dataset. In the case of C5 gene datasets, all the methods tied, each obtaining the largest (9/10 gene sets) among all the ranking methods. As the four datasets are obtained from two RNA-seq platforms, Illumina and SOLiD, DNMF achieved higher consistence of gene sets than that of the compared methods in balance, although all methods performed well.

**Table 2:** Overlap of the top10 gene sets enriched by 6 different gene-ranking methods on four datasets for the C2CP, C2KEGG and C5 gene datasets.

	DESeq	DNMF	edgeR	gfold	gfoldFC	PossionSeq
C2CP	8	9	8	7	9	8
C2KEGG	7	8	8	6	7	7
C5	9	9	9	9	9	9

We also evaluated the biological content of enriched gene sets. S1 Table details the consistent gene sets obtained by DNMF on the C2CP, C2KEGG and C5 gene sets. Pooled from several brain regions, the enriched gene sets for sample group B should be relevant to brain-function. As we can see from the canonical pathway dataset (C2CP) in S1 Table, reactome of neuronal system, chemical synapses transmission, olfactory, neural related function in postsynaptic cell, and neurotransmitter release cycle are enriched. Furthermore, potassium channel [29] and calcium signalling are also enriched. Similar results are found in the KEGG gene set. For biological process within Gene Ontology (C5), neural related processes, such as nervous system development, neurological system process, nerve impulse and potassium ion transport, are enriched. From S1 Table, we can conclude that the overlapped gene sets are highly related with the phenotype of the sample.

**Fig 4:**
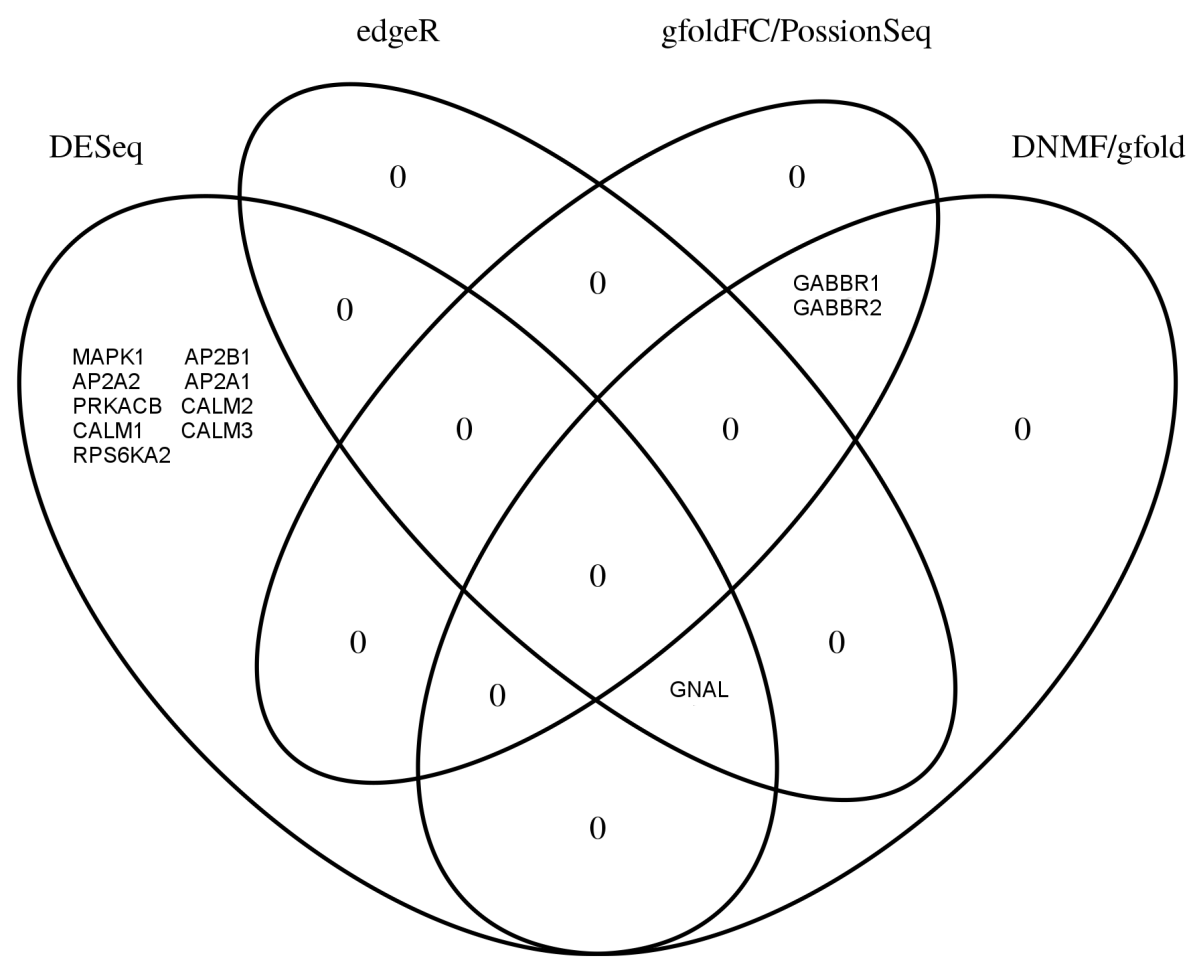
Venn diagram of leading edge genes among the leading edge subsets for all methods. The leading edge genes are highly overlapped from methods except DESeq. All the leading edge genes are relevant to brain-function.

Next, we analysed by GSEA the leading edge subset of the top 10 C2CP gene set for sample B in the AGR dataset. Genes in many of the leading edge subsets are more likely to be interesting than genes that are only seen in a few of the leading-edge subsets [2]. As a result, genes seen most frequently in the top 10 gene sets were obtained (see Fig 4). No gene was present in all the 10 gene sets. The leading edge genes identified by all the methods except DESeq are similar. More specifically, three leading edge genes (GNAL, GABBR1 and GABBR2) obtained by DNMF and gfold are involved in neuroactive ligand receptor interaction pathway from KEGG [30]. At the same time, leading edge genes identified by DESeq are relevant with Long-term potentiation (LTP) pathway from KEGG. LTP is one of the major cellular mechanisms that underlies learning and memory [31].

### Comparison of computation time

We tested the runtime of all compared methods for ranking genes on the NWU dataset on an 8-core 3.4GHz, 16GB RAM, Debian desktop PC. DNMF only spent 0.78 seconds, while edgeR took 3.94 seconds, DESeq 2.22 minutes, PoissonSeq 3.68 seconds, gfold 482.94 seconds and DESeq 133.2 seconds. Moreover, in order to check the scalability of DNMF on larger datasets, we used the original NWU dataset with 120 samples from different lanes and flowcells. Only 3.37 seconds were spent on this large dataset. The efficiency of DNMF largely results from NMF itself and specific initiation of H in DNMF we used. Consequently, DNMF runtime substantially outperformed other methods and scaled well on large datasets.

## Discussion

We leverage Discriminant Non-negative Matrix Factorization (DNMF), a machine learning method, to rank genes identified by RNA-seq analysis. This paper evaluates the performance of our proposed DNMF-based gene ranking method by comparing with the state-of-the-art methods including DESeq, edgeR, PoissonSeq, gfoldFC and gfold, across a range of analytical perspectives. We conclude that DNMF performs well in a number of aspects. Firstly, gene ranking by DNMF is less affected by gene expression levels. Although Fisher criterion is used in DNMF and subtraction is used in the difference between-class, we logged gene expression data so that division is used in the difference between-class in unlogged data. The log operation for DNMF also results in almost no bias towards genes with larger read counts. Secondly, theoretically, gene ranking can be considered as an extension of differential expression analysis. We tested the performance of DNMF on the benchmarked differentially expressed genes defined by both ERCC spike-ins and qPCR validated data. Accordingly, we employ AUC curves to evaluate different ranking methods. DNMF consistently outperforms the representative methods in most cases, indicating a high sensitivity and specificity. Thirdly, the performance of gene set enrichment analysis is largely dependent on the relative rank of genes. As the four datasets involve samples from directly comparable tissues, we selected the top 10 enriched gene sets from the results of all four datasets sourcing from each ranking method to test the consistency between the enriched gene sets. Compared with others, the proposed DNMF-based ranking method shows a high degree of consistency between the enriched gene sets based on three gene datasets. Moreover, the enriched gene sets are biologically related with the sample. Collectively, we believe the comparisons demonstrate that DNMF may provide a more biologically meaningful gene rank resulting in more robust gene sets and pathways for further investigation.

The reason why DNMF outperforms other methods probably resides in three aspects. Firstly, depending on the objective function used, DNMF appears to be quite robust to noise within data, even after incorporating Fisher’s criterion with an accompanying disruption to the originally modelled noise distribution of the data set. Secondly, DNMF can efficiently and effectively identify the up-regulated and down-regulated metagenes, benefitting from the non-negative constraints, Fisher’s criterion, subtle initiation of H and the simplified selection of two dimensions. With different weight assigned to different genes, the two metagenes play a critical role in gene ranking. In addition, in a similar manner to NMF, DNMF has potential to learn sparse and parts-based representation (such as a nose on a facial image), and therefore DNMF can differently weight genes that potentially play different roles in different kinds of samples, as evidenced by sample specific expression behaviours. As the weight of genes will have a high impact on the gene ranking and on the power of the subsequent tests, DNMF can identify a biologically and statistically meaningful rank of genes.

DNMF still has a theoretical shortcoming that it cannot always identify the up-regulated and down-regulated metagenes when there is a convergence to a stationary limit point. Thus, if the coefficient H does not represent the up-regulated and down-regulated metagenes, a rerun of DNMF is required until the condition satisfies. Note that our DNMF source code will guide the user to perform this operation if required. At the same time, as a differential expression analysis tool, DNMF cannot deal with multi-factored experiments like edgeR or DESeq or experiments without replicates like gfold.

We have developed a simple DNMF package to enable convenient gene ranking in gene expression experiments. The tolerable computational cost of the algorithm makes it competitive with other more intensively computational methods. DNMF provides an effective gene-ranking method, and is also extensible to other genes ranking functions, such as rank-based similarity measures for gene expression data [32], besides GSEA. Furthermore, DNMF can be used as a differential expression analysis tool by selecting the top genes in the up-regulated and down-regulated metagenes in a similar manner to that used by Kong et al [11]. DNMF may also be applicable for the large scale meta-analyses of gene expression data measured on different platforms towards a consensus on gene sets in key diseases and biological traits. The DNMF approach could be generically applied to other fields allied to and beyond biology, such as the challenging and high dimensional interface between chemistry and biology which is often a barrier during the process of drug discovery. In conclusion, we have shown a novel application of DNMF for gene ranking of RNA-seq gene expression data, which greatly outperforms other representative methods in benchmark testing.

## Supporting Information

S1 Fig MA plot of top 1000 differentially expressed genes in the BGI dataset identified by 6 methods. (TIFF)

S2 Fig MA plot of top 1000 differentially expressed genes in the NWU dataset identified by 6 methods. (TIFF)

S3 Fig MA plot of top 1000 differentially expressed genes in the PSU dataset identified by 6 methods. (TIFF)

S4 Fig MA plot of top 10 differentially expressed genes in the AGR dataset identified by 6 methods. (TIFF)

S1 Table The overlapping gene sets by DNMF for the four datasets using C2CP, C2KEGG and C5 gene datasets. Most of them are related with brain-function.
(DOCX)
