# Supplementary figures and images for: Gene Ranking of RNA-Seq Data via Discriminant Non-Negative Matrix Factorization
Source: PLoS One. 2015 Sep 8;10(9):e0137782. doi: 10.1371/journal.pone.0137782 (PMC4562600; doi:10.1371/journal.pone.0137782)

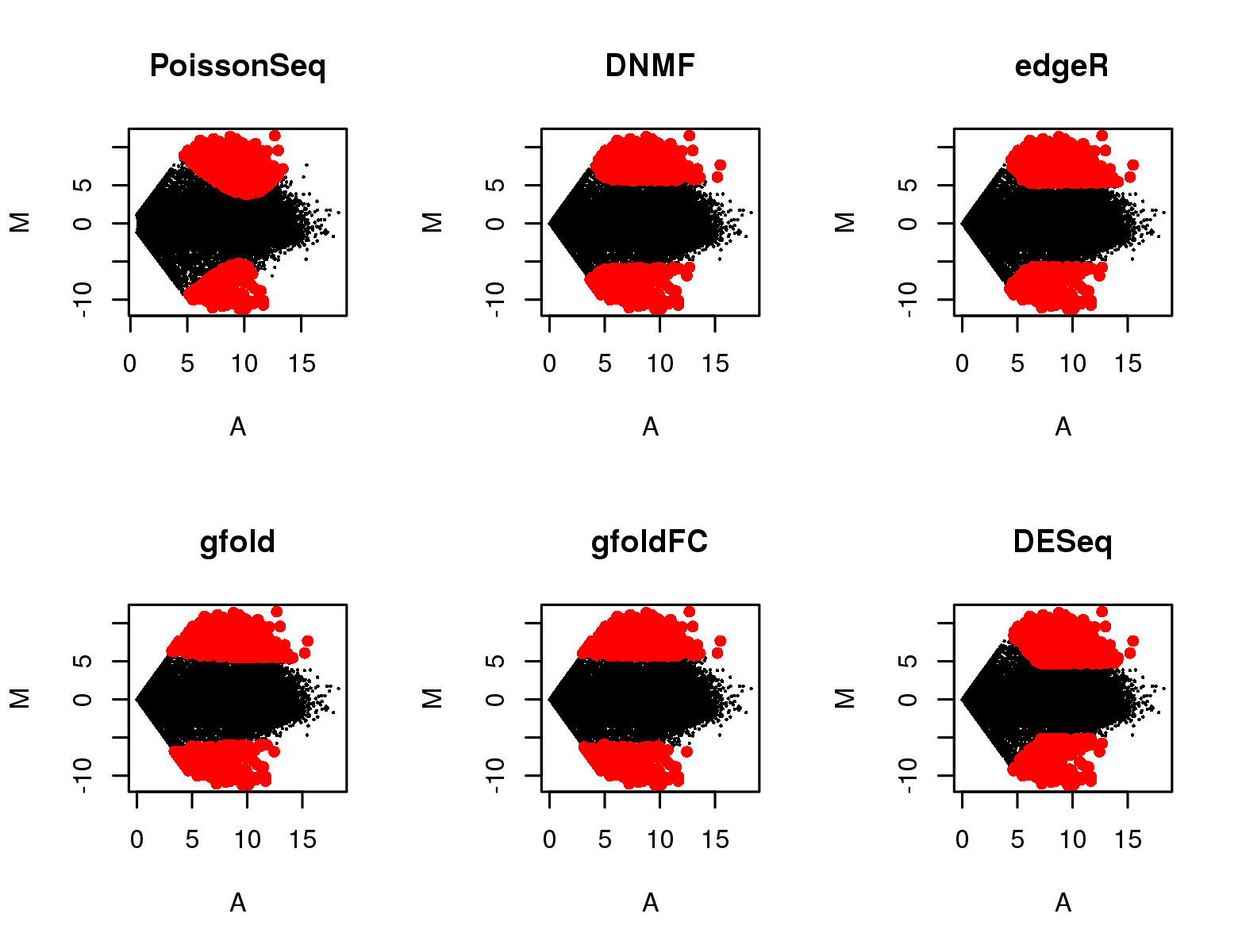

Supplement: S1 Fig — (TIFF) [file pone.0137782.s001.tiff]

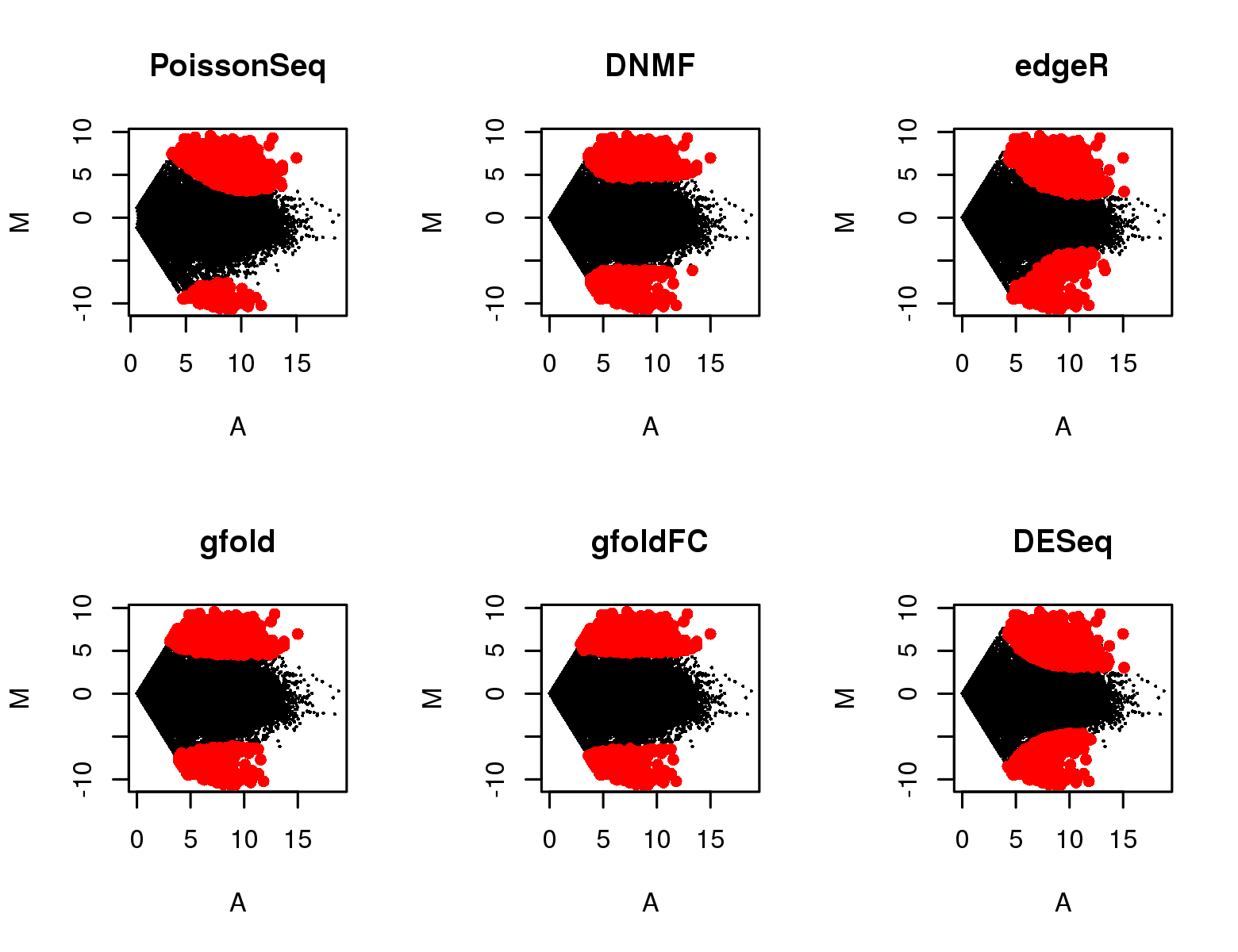

Supplement: S2 Fig — (TIFF) [file pone.0137782.s002.tiff]

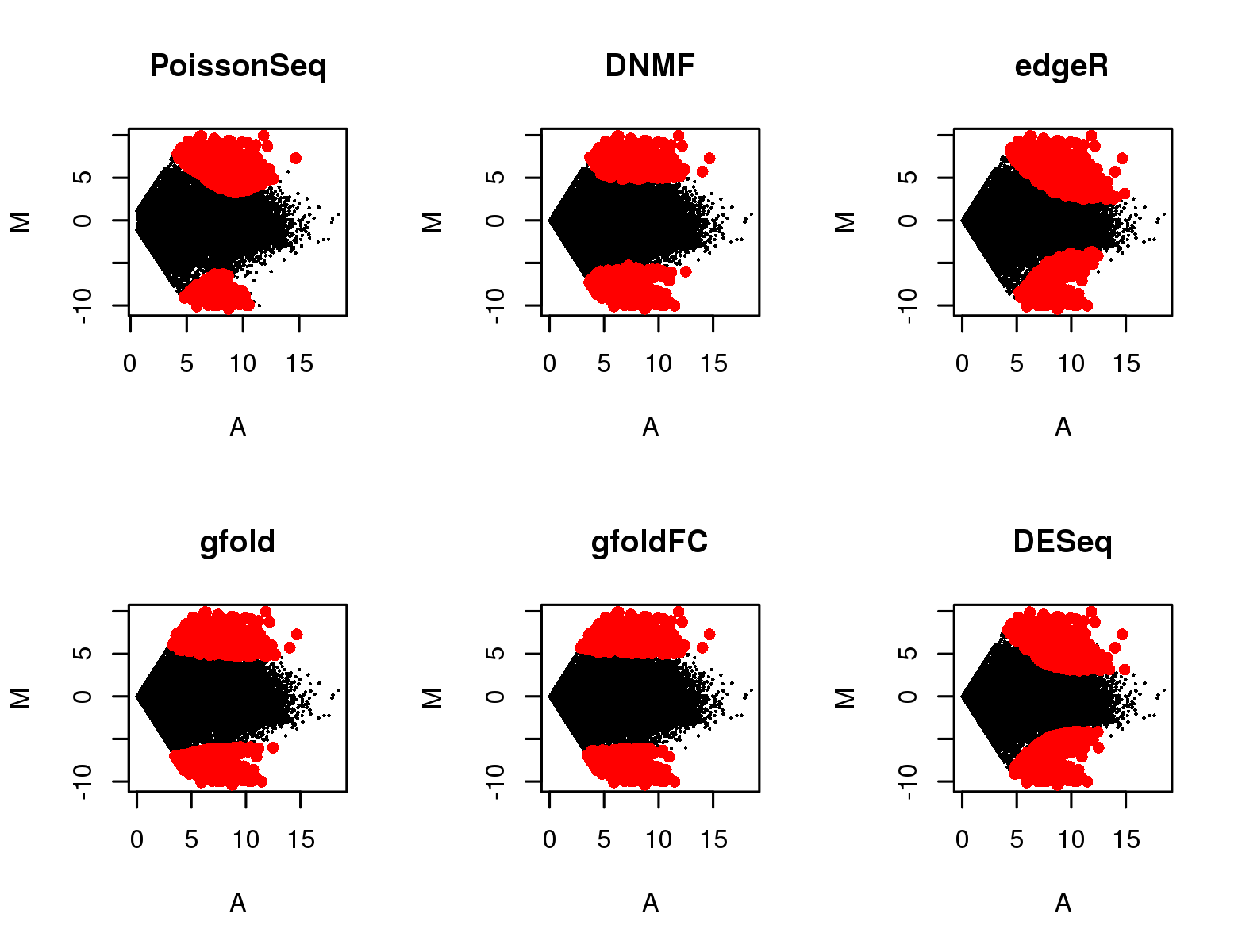

Supplement: S3 Fig — (TIFF) [file pone.0137782.s003.tiff]

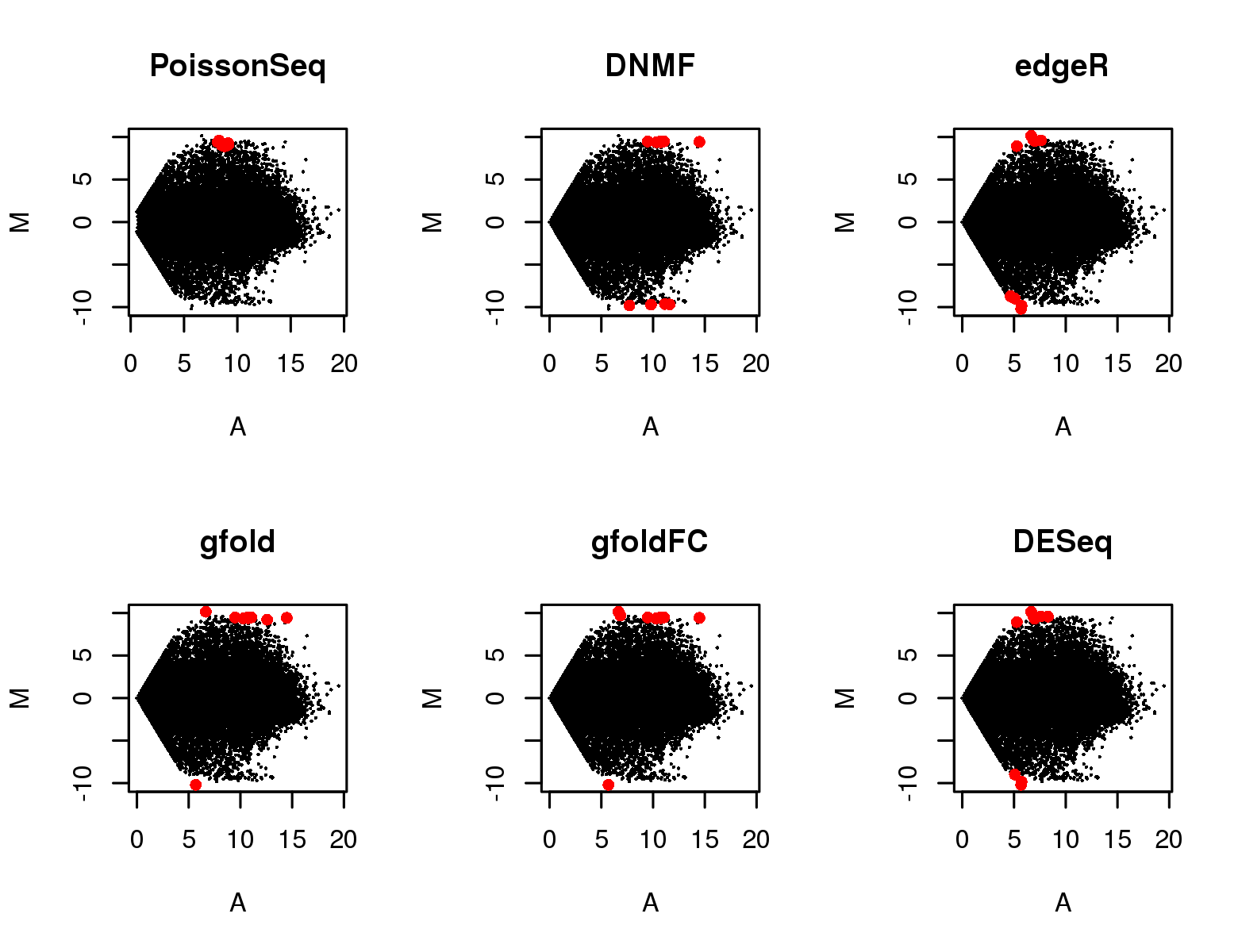

Supplement: S4 Fig — (TIFF) [file pone.0137782.s004.tiff]
