# Supplementary material for: Gene Ranking of RNA-Seq Data via Discriminant Non-Negative Matrix Factorization
Source: PLoS One. 2015 Sep 8;10(9):e0137782. doi: 10.1371/journal.pone.0137782 (PMC4562600; doi:10.1371/journal.pone.0137782)
Supplement: S1 Table — Most of them are related with brain-function. (DOCX) [file pone.0137782.s005.docx]

­­Gene Ranking of RNA-seq data via Discriminant Non-negative Matrix Factorization

Supporting information

**S1 Table. The overlapping gene sets by DNMF for the four datasets using C2CP, C2KEGG and C5 gene datasets.** Most of them are related with brain-function.

| **C2CP** | REACTOME_NEURONAL_SYSTEM  REACTOME_TRANSMISSION_ACROSS_CHEMICAL_SYNAPSES  KEGG_OLFACTORY_TRANSDUCTION  REACTOME_OLFACTORY_SIGNALING_PATHWAY  REACTOME_NEUROTRANSMITTER_RECEPTOR_BINDING_AND_DOWNSTREAM_TRANSMISSION_IN_THE_POSTSYNAPTIC_CELL  KEGG_NEUROACTIVE_LIGAND_RECEPTOR_INTERACTION  REACTOME_POTASSIUM_CHANNELS  KEGG_CALCIUM_SIGNALING_PATHWAY  REACTOME_NEUROTRANSMITTER_RELEASE_CYCLE |
| --- | --- |
| **C2KEGG** | KEGG_OLFACTORY_TRANSDUCTION  KEGG_NEUROACTIVE_LIGAND_RECEPTOR_INTERACTION  KEGG_CALCIUM_SIGNALING_PATHWAY  KEGG_PHOSPHATIDYLINOSITOL_SIGNALING_SYSTEM  KEGG_LONG_TERM_POTENTIATION  KEGG_AXON_GUIDANCE  KEGG_VASCULAR_SMOOTH_MUSCLE_CONTRACTION  KEGG_CELL_ADHESION_MOLECULES_CAMS |
| **C5** | NERVOUS_SYSTEM_DEVELOPMENT  NEUROLOGICAL_SYSTEM_PROCESS  SYNAPTIC_TRANSMISSION  TRANSMISSION_OF_NERVE_IMPULSE  CELL_CELL_SIGNALING  METAL_ION_TRANSPORT  POTASSIUM_ION_TRANSPORT  CATION_TRANSPORT  ION_TRANSPORT |
